# Comparative transcriptome and metabolome analysis reveal key regulatory defense networks and genes involved in enhanced salt tolerance of *Actinidia* (kiwifruit)

**DOI:** 10.1093/hr/uhac189

**Published:** 2022-08-25

**Authors:** Muhammad Abid, Shichao Gu, Yong-Jie Zhang, Shihang Sun, Zhi Li, Dan-Feng Bai, Leiming Sun, Xiu-Juan Qi, Yun-Peng Zhong, Jin-Bao Fang

**Affiliations:** Key Laboratory for Fruit Tree Growth, Development and Quality Control, Zhengzhou Fruit Research Institute, Chinese Academy of Agricultural Sciences, Zhengzhou 450009, China; Lushan Botanical Garden, Chinese Academy of Sciences, Jiujiang 332900, China; Key Laboratory for Fruit Tree Growth, Development and Quality Control, Zhengzhou Fruit Research Institute, Chinese Academy of Agricultural Sciences, Zhengzhou 450009, China; Key Laboratory for Fruit Tree Growth, Development and Quality Control, Zhengzhou Fruit Research Institute, Chinese Academy of Agricultural Sciences, Zhengzhou 450009, China; Key Laboratory for Fruit Tree Growth, Development and Quality Control, Zhengzhou Fruit Research Institute, Chinese Academy of Agricultural Sciences, Zhengzhou 450009, China; Key Laboratory for Fruit Tree Growth, Development and Quality Control, Zhengzhou Fruit Research Institute, Chinese Academy of Agricultural Sciences, Zhengzhou 450009, China; Key Laboratory for Fruit Tree Growth, Development and Quality Control, Zhengzhou Fruit Research Institute, Chinese Academy of Agricultural Sciences, Zhengzhou 450009, China; Key Laboratory for Fruit Tree Growth, Development and Quality Control, Zhengzhou Fruit Research Institute, Chinese Academy of Agricultural Sciences, Zhengzhou 450009, China; Key Laboratory for Fruit Tree Growth, Development and Quality Control, Zhengzhou Fruit Research Institute, Chinese Academy of Agricultural Sciences, Zhengzhou 450009, China; Key Laboratory for Fruit Tree Growth, Development and Quality Control, Zhengzhou Fruit Research Institute, Chinese Academy of Agricultural Sciences, Zhengzhou 450009, China; Key Laboratory for Fruit Tree Growth, Development and Quality Control, Zhengzhou Fruit Research Institute, Chinese Academy of Agricultural Sciences, Zhengzhou 450009, China

## Abstract

The *Actinidia* (kiwifruit) is an emerging fruit plant that is severely affected by salt stress in northern China. Plants have evolved several signaling network mechanisms to cope with the detrimental effects of salt stress. To date, no reported work is available on metabolic and molecular mechanisms involved in kiwifruit salt tolerance. Therefore, the present study aims to decipher intricate adaptive responses of two contrasting salt tolerance kiwifruit species *Actinidia valvata* [ZMH (an important genotype), hereafter referred to as R] and *Actinidia deliciosa* [‘Hayward’ (an important green-fleshed cultivar), hereafter referred to as H] under 0.4% (w/w) salt stress for time courses of 0, 12, 24, and 72 hours (hereafter refered to as h) by combined transcriptome and metabolome analysis. Data revealed that kiwifruit displayed specific enrichment of differentially expressed genes (DEGs) under salt stress. Interestingly, roots of R plants showed a differential expression pattern for up-regulated genes. The KEGG pathway analysis revealed the enrichment of DEGs related to plant hormone signal transduction, glycine metabolism, serine and threonine metabolism, glutathione metabolism, and pyruvate metabolism in the roots of R under salt stress. The WGCNA resulted in the identification of five candidate genes related to glycine betaine (GB), pyruvate, total soluble sugars (TSS), and glutathione biosynthesis in kiwifruit. An integrated study of transcriptome and metabolome identified several genes encoding metabolites involved in pyruvate metabolism. Furthermore, several genes encoding transcription factors were mainly induced in R under salt stress. Functional validation results for overexpression of a candidate gene *betaine aldehyde dehydrogenase* (*AvBADH,* R_transcript_80484*)* from R showed significantly improved salt tolerance in *Arabidopsis thaliana* (hereafter referred to as *At*) and *Actinidia chinensis* [‘Hongyang’ (an important red-fleshed cultivar), hereafter referred to as *Ac*] transgenic plants than in WT plants. All in all, salt stress tolerance in kiwifruit roots is an intricate regulatory mechanism that consists of several genes encoding specific metabolites.

## Introduction

Fruit plants play a promising role in human lives due to their nutritional benefits for health. Abiotic stresses cause a massive loss in fruit crop production by restricting their growth and development. Most of the fruit plants, due to varying ability of salt tolerance, are severely damaged by salt stress due to being perennials. To withstand the damaging effects of salt stress, plants have evolved several tolerance mechanisms. Many genes have been induced or suppressed in fruit plants under salt stress [1–3]. The decreased cultivable area due to rising sea level, decrease in groundwater recharge, traditional irrigation methods, and increased evapotranspiration is a major concern worldwide [4]. Therefore, it is highly prudent to identify metabolic pathways and salt stress-responsive genes to improve fruit plant genetic resources that can thrive well in salt-affected soils.

Plant survival in adverse environmental conditions is based on integrating stress-adaptive metabolic and structural changes in endogenous developmental programs [5]. The complex processes of regulation of salt tolerance in plants and salt adaptation involve the control of water flux and osmotic adjustments at the cellular level through osmoprotectant biosynthesis [6]. Whereas ionic stress caused by salt copes with controlled influx and efflux of ions at the plasma membrane and sequestering of the ions in the vacuole [5]. Similarly, the imbalanced redox homeostasis and energy supply in plants are regulated by reprogramming the metabolism and impaired cellular structure [7]. Various functional genes and regulatory genes control these regulatory events. Therefore, it is imperative to understand the role of metabolic pathways and complex defense-related signaling networks involved in salt stress tolerance in plants.

Generally, the salt tolerance in plants is associated with the accumulation of specific metabolites [8] and the number of genes involved in the biosynthesis of these metabolites [9, 10]. Some of these genes are *BADH* [11], *NHX1* [12], *AISP* [13], *P5CS* [14], *CCD1* [15], *WRKY8* [16], *NAC06* [17], *ERF11* [18], and *MADS25* [19]. Similarly, some important primary and secondary metabolites for salt stress tolerance are sugars, polyols, amino acids [20, 21], nitrogen-containing compounds, terpenes, and phenolic compounds [22]. Recently, high-throughput omics technologies, i.e. transcriptome and metabolome, have been developed, which are critical in identifying the regulatory networks and metabolic reorganizations for controlling salinity tolerance in plants [23]. To date, several integrated studies of transcriptome and metabolome have been reported in some plants against salt stress [23–26].

The *Actinidia*, commonly known as kiwifruit, is a recently adopted fruit plant gaining popularity among customers due to its unique flavor and nutritional benefits. It is comprised of 54 species and 21 varieties, a total of 75 taxa [27, 28]. However, most of the available commercial kiwifruit cultivars are salt-sensitive. Additionally, there is no reported work available on transcriptome and metabolome analysis in kiwifruit for salt stress so far. Previously, we screened different kiwifruit genotypes to assess their tolerance ability against salt stress [29, 30]. Based on previous results, the compoarative analysis of transcriptome and metabolome was conducted to decipher the intricate metabolic pathways and their regulatory gene network involved in conferring salt tolerance in kiwifruit. The data obtained from the present study will facilitate researchers to identify salt stress-responsive networks in kiwifruit. Additionally, it will deepen the understanding of the salt resistance mechanism in fruit trees and accelerate the cultivation of salt-tolerant varieties in kiwifruit.

## Results

### Performance of R and H for salt stress tolerance

**Figure 1:**
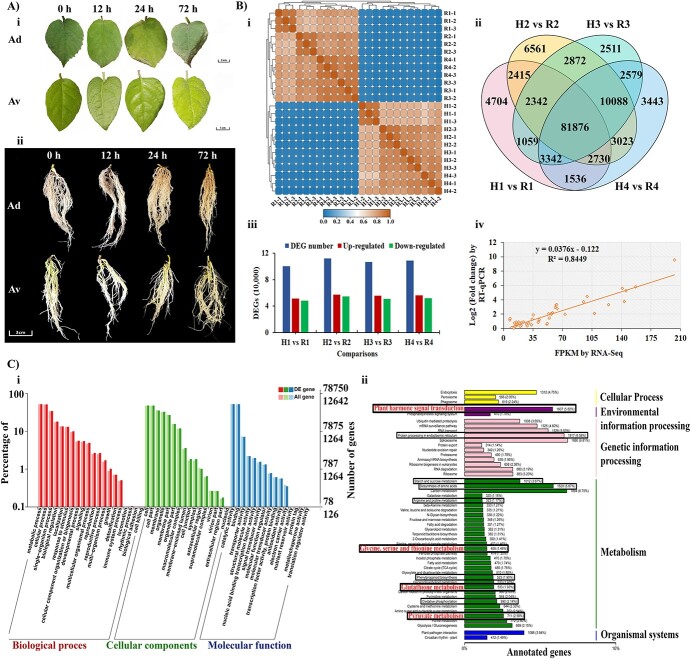
Morphological changes of R and H, an overview of RNA-seq data, and validation of DEGs expression pattern in R and H under 0.4% NaCl stress. (**A**) Morphological changes of R and H in (**i**) leaves and (**ii**) roots. (**B**) Overview of RNA-seq data (**i**) expression-dependent heatmap of samples obtained from R and H by using data from spearman correlation coefficient analysis. The x-axis and y-axis represents samples taken from *Av* and H under salt stress, (**ii**) statistics of commonly up-regulated genes in R and H, (**iii**) statistics of up-regulated and down-regulated DEGs in roots, and (**iv**) validation of RNA-seq data by RT-qPCR analysis. (**C**) GO terms and KEGG enrichment analysis of up-regulated genes in R4 vs H4. (**i**) GO terms enrichment analysis. The x-axis represents enriched GO terms and y-axis represents the number of up-regulated genes in a particular GO term. The red columns are for GO terms under biological process, the Green color represents GO terms under cellular components and the blue color stands for GO terms in molecular function. (**ii**) KEGG enrichment analysis. Vertical columns represent enriched pathways and horizontal columns are for annotated genes. R, *Actinidia valvata*; H, *A. deliciosa*; h, hours.

In our previous study, we reported the varying tolerance ability of R and H against salt stress [29]. In the present study, we put efforts to assess the salt stress tolerance of R and H at different time courses. Plants were treated with 0.4% NaCl (w/w) for 12 h, 24 h, and 72 h by keeping 0 h (untreated) plants as control. The leaves of the H plants showed noticeable wilting and necrosis compared with R plants (Fig. 1Ai). Similarly, the roots of the H plants turned into brownish color under salt stress while no visible evidence for salt stress damage was found in R roots (Fig. 1Aii).

### Overview of transcriptome profiling and validation of RNA-seq data by qPCR

A global transcriptional profile of roots sampled from R and H treated with or without salt stress was carried out to thoroughly examine the intricate mechanisms involved in enhanced salt stress tolerance. For convenience, R and H were used to represent ZMH from *Actinidia valvata* and ‘HWD’ from *A. deliciosa*, while 1, 2, 3, and 4 represent 0 h, 12 h, 24 h, and 72 h, respectively. The statistics for PacBio and Illumine Hi-seq transcriptome sequencing are presented in the online supplementary material (Table S1). The hierarchical clustering of 24 samples by using Spearman correlation coefficient analysis data showed that all samples from H and R were clustered in clade one and clad two, respectively (Fig. 1Bi). It signifies that the gene expression profile for samples was consistent. A pairwise comparison method was adopted to identify DEGs responsive to salt stress. We identified 100 004 DEGs for R1 vs H1, 111 907 DEGs for R2 vs H2, 106 669 DEGs for R3 vs H3, and 108 617 DEGs for R4 vs H4 (Fig. 1Bii, iii, and Fig. S1, see online supplementary material). Based on the phenotype results (Fig. 1A) and the total number of common DEGs (Fig. 1B, ii, iii, and Fig. S1, see online supplementary material), we selected the R4 vs H4 comparison for further analysis. We did RNA-seq data validation by checking the relative expression of 16 randomly selected genes with RT-qPCR (Fig. S2, see online supplementary material). The detail for the pair of primers used for RT-qPCR are given in the supplementary section (Table S2, see online supplementary material). The scatter plot of 16 genes from RT-qPCR and RNA-seq data showed a linear trend (Fig. 1Biv). The higher correlation (R^2^ = 0.8449) between RT-qPCR and RNA-seq results implied that our RNA-seq data is reliable for further assessment.

### Differentially expressed salt stress-responsive genes

To find the salt stress-responsive DEGs for kiwifruit, the GO enrichment analysis and KEGG analysis for DEGs in R4 vs H4 were carried out. The GO enrichment analysis provided additional information about the key processes and pathways that distinguished the response of R and H against salt stress. This analysis resulted in identification of 126 422 GO terms in which 78 750 were statistically significant under biological process, cellular process, and molecular function. The most prominent GO terms that were commonly found in roots included metabolic process, cellular processing, cell, cell parts, catalytic activity, binding, response to stimulus, metabolic process, signaling, detoxification, signal transducer activity, antioxidant activity, etc. (Fig. 1Ci).

According to KEGG analysis, 10 out of the top 50 pathways were specifically identified based on their relevance to salt stress tolerance. Among these 10 pathways, most numbers of annotated DEGs were linked to protein processing (1817, 6.56%), biosynthesis of amino acids (1620, 5.87%), plant hormone signal transduction (1607, 5.82%), starch and sucrose metabolism (101, 3.67%), pyruvate metabolism (711, 2.58%), oxidative phosphorylation (590, 2.14%), glutathione metabolism (533, 1.93%), phenylpropanoid biosynthesis (523, 1.90%), glycine, serine, and threonine metabolism (409, 1.48%), and arginine and proline metabolism (323, 1.17%) (Fig. 1Cii).

### Important pathways conferring salt stress tolerance

Salt stress can lead to over-accumulation of various metabolites in plants. The salt tolerance ability of plants is typically assessed by the maintenance of proper levels of metabolic processes and defense responses. We carried out comprehensive study for important metabolic KEGG pathways to find out the reason behind the elevated salt tolerance ability of the R plants. We selected glutathione metabolism pathway [31–33], pyruvate metabolism pathway [34, 35], glycine betaine metabolism pathway [36–38], ABA signaling pathway [39, 40], and MYB transcription factors [41, 42] due to their important role in conferring salt tolerance in plants that is extensively reported by researchers in the past (Fig. 1Cii).

### ABA signaling was altered in R under salt stress

**Figure 2:**
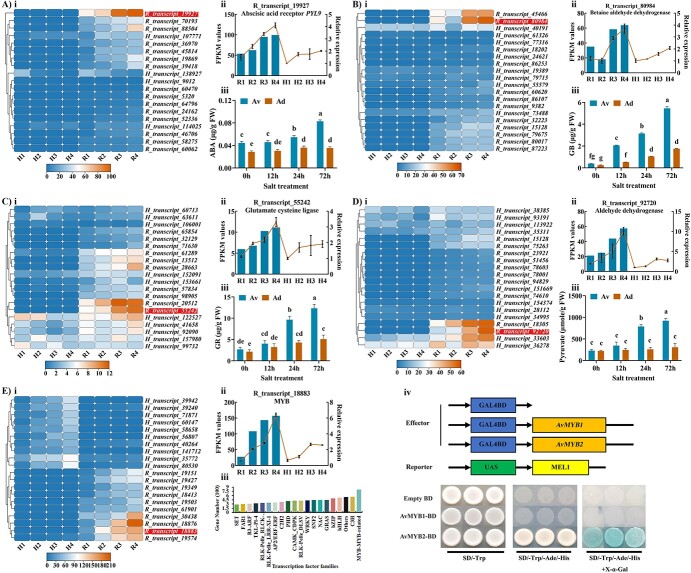
Important pathways conferring salt stress tolerance in kiwifruit. (**A**) Analysis of the ABA pathway. (**i**) Heatmap for top 20 up-regulated DEGs. (**ii**) The expression profile of identified candidate gene R_transcript_19927 (*Abscisic acid receptor PYL9*). (**iii**) Contents of abscisic acid (ABA) hormone. (**B**) Analysis of glycine betaine (GB) pathway. (**i**) Heatmap of top 20 up-regulated DEGs. (**ii**) The expression profile analysis of candidate gene R_transcript_80984 (*Betaine Aldehyde dehydrogenase*). (**iii**) Contents of glycine betaine (GB). (**C**) Analysis of glutathione metabolism pathway. (**i**) Heatmap for top 20 up-regulated DEGs. (**ii**) The expression profile for candidate gene R_Transcript_55242 (*Glutamate cysteine ligase*). (**iii**) Contents of glutathione. (**D**) Analysis of pyruvate biosynthesis pathway. (**i**) Heatmap for 20 up-regulated DEGs. (**ii**) The expression profile for candidate gene R_transcript_92720 (*Aldehyde dehydrogenase*). (**iii**) Contents of pyruvate. (**E**) Analysis of transcription factors (TFs) families*.* (**i**) Heatmap for top 20 MYB family genes. (**ii**) The expression profile of candidate gene R_transcript_ 18 883 (MYB transcription factor). (**iii**) the Total number of transcription factors (TF) in each annotated family. (**iv**) Transcriptional activation activity analysis for MYB1 (R_transcript_18876) and MYB2 (R_transcript_18 883) TFs. Each value represents the mean ± SD of three biological replicates. Full names for abbreviations are presented in the online supplementary material (Table S3).

In the plant hormone signal transduction pathway, a total of 1607 annotated genes were identified among which 833 were up-regulated DEGs. These up-regulated genes belong to Auxin (176 genes), ABA (112 genes), CTK (113 genes), BR (110 genes), Ethylene (106 genes), GA (39 genes), JA (77 genes), and SA (100 genes) (Fig. S3, see online supplementary material). As the role of ABA in salt stress is well reported previously, we did a comprehensive analysis of ABA signaling in kiwifruit. The expression pattern of the top 20 DEGs for both species under 0.4% salt stress is presented in the heatmap (Fig. 2Ai). The R_transcript_19927 (Abscisic acid receptor *PYL9*) was found to play role in the tolerance mechanism as its expression was significantly higher in R compared to H. After performing RT-qPCR, we obtained a similar expression pattern for R_transcript_19927 as in RNA-seq data (Fig. 2Aii). As additional evidence, we measured the ABA contents in kiwifruit. Surprisingly, the trend for change in contents was identical to gene expression results. The ABA contents in R significantly increased over time, while no significant change was found in H (Fig. 2Aiii).

### The R showed enhanced accumulation of glycine betaine than H

For the GB pathway, a total of 246 up-regulated DEGs were identified in R4 vs H4 comparison. According to the heatmap of FPKM values, we found that the expression pattern of R_transcript_45466 and R_transcript_80984 was significantly different in R than in H under salt stress (Fig. 2Bi). Both transcripts are involved in the biosynthesis of GB and annotated as *BADH* (*betaine aldehyde dehydrogenase*). We validated the gene expression results of R_transcript_80984 (*betaine Aldehyde dehydrogenase*) from RNA-seq by RT-qPCR and found both results consistent (Fig. 2Bii). It indicated that R possessed a different salt-induced GB pathway than H. To confirm our assumption, we measured GB contents in both species under the same salt stress. The results showed that R accumulated noticeably higher GB than H (Fig. 2Biii).

### Glutathione conferred salt stress tolerance in R plants

Elevated salt stress conditions lead to an increase in the accumulation of ROS in plants. The KEGG analysis identified glutathione metabolism among the top 50 differentially expressed KEGG pathways (Fig 4). The expression profile for the top 20 DEGs in both species under salt stress is presented in the heatmap (Fig. 2Ci). Many transcripts were differentially expressed between R and H plants. The expression pattern for R_transcript_55242 (Glutamate cysteine ligase) was analysed based on FPKM values and validated by RT-qPCR (Fig. 2Cii). These results provided clues about the involvement of glutathione in the enhanced tolerance ability of R plants. We measured glutathione contents in both species under salt stress as evidence to prove our assumption. The R showed a significantly enhanced accumulation of glutathione compared with H (Fig. 2Ciii).

### The R accumulated more pyruvate than H

Pyruvate metabolism provides energy backup for plants to efficiently cope with the catastrophic effects of salt stress. In our analysis, pyruvate fell among the top 50 differentially expressed KEGG pathways (Fig. 4). There were in total 357 up-regulated DEGs in this pathway. Expression profiles of the top 20 DEGs were clearly different in R and H under salt stress (Fig. 2Di). The expression profile for R_transcrip_92720 (Aldehyde dehydrogenase) was analysed and validated by FPKM and RT-qPCR, respectively (Fig. 2Dii). By measuring the contents of pyruvate, we found that R accumulated a significantly higher amount of pyruvate compared with H (Fig. 2Diii).

### Transcription factors were differentially expressed in R and H under salt stress

Transcription factors are responsible for the regulation of downstream genes, and they function in multiple biological processes. In the case of our study, salt stress induced many TFs with differential expression patterns between R and H. We found 20 TF families that were prominently expressed in kiwifruit under salt stress (Fig. 2Eiii). The MYB family was deliberately picked based on the abundance of gene numbers. The expression pattern of the top 20 DEGs showed a clear difference between R and H (Fig. 2Ei). Furthermore, the expression pattern of candidate gene R_transcript_ 18 883 (MYB transcription factor) by FPKM analysis and qPCR showed that it was specifically expressed in R under salt stress (Fig. 2Eii). The results from transcriptional activation activity showed that *AvMYB2* (R_transcript-18 883) had ability to activate expression of downstream reporter gene (Fig. 2Eiv).

### Identification of regulatory networks and hub genes

**Figure 3:**
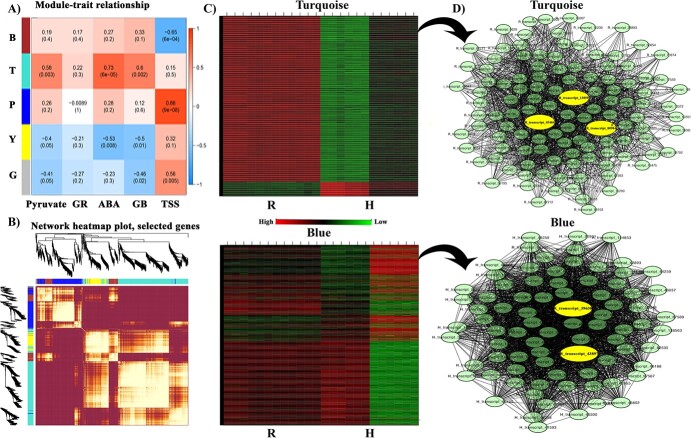
Identification of gene networks and key candidate genes involved in enhanced salt tolerance of kiwifruit. (**A**) Correlation plot representing the trait-modules relationship. (**B**) Heatmap plot of gene networks. Light colors represent low overlapping and dark color represents high overlapping of gene pairs. (**C**) Heatmaps for selected color modules. (**D**) Gene network for the blue and turquoise module. ABA, abscisic acid; B, blue; G, grey; GB, glycine betaine; GR, glutathione reductase; H, *A. deliciosa*; R, *Actinidia valvata*; T, turquoise; TSS, total soluble sugars; Y, yellow.

The WGCNA analysis was implied on transcriptome data which identified five distinct color modules based on the co-expression pattern of individual genes presented in the network heatmap plot (Fig. 3B). The clustered dendrogram of DEGs from R and H is presented in the online supplementary material (Fig. S4). The ABA, TSS, glutathione, pyruvate, and GB contents in R and H under salt stress were used as phenotypic input data to carry out module-trait relationship analysis and results are presented in the module-trait relationship plot (Fig. 3A). Out of five color modules, the blue module showed a significant correlation with TSS, while the turquoise model was significantly correlated with pyruvate, GB, and ABA. The turquoise module contained 1057 genes that showed a significant association with pyruvate (R^2^ = 0.58), ABA (R^2^ = 0.73), and GB (R^2^ = 0.6). Similarly, the blue module comprised 354 genes that showed a significant relationship with TSS and GR (R^2^ = 0.86). The expression profile for all the genes used in turquoise and blue color modules is presented in heatmaps (Fig. 3C). The genes from both modules were selected for their edges and nodes calculation which were later used for gene-network visualization purpose. This analysis resulted in the identification of two genes annotated as *betaine aldehyde dehydrogenase* (R_transcript_45466 and R_transcript_80984) and one gene annotated as *pyruvate kinase* (R_transcript_13859) in the turquoise color module (Fig. 3D, upper panel). Similarly, the blue module resulted in the identification of hub genes related to TSS annotated as *fructokinase* (H_transcript_39638) and another gene related to glutathione annotated as *glutathione-s-transferase* (H_transcript_43897) (Fig. 3D, lower panel). The changes in contents of TSS are presented in the online supplementary material) (Fig. S5). All identified hub genes are highlighted as yellow color in the network plot.

### Overview of metabolome profile in R and H under salt stress

**Figure 4:**
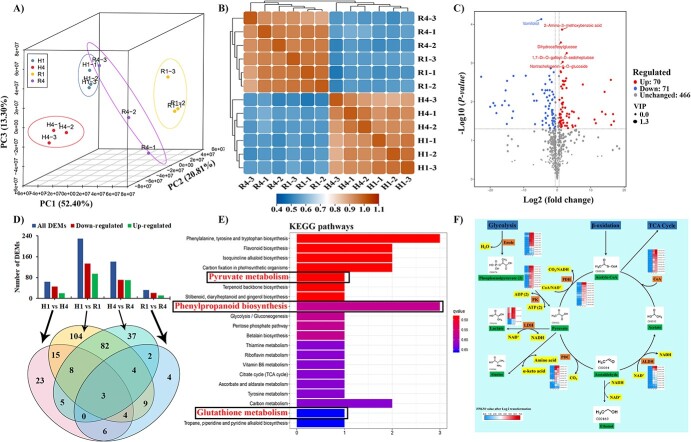
Overview for metabolome data and integrated metabolome and transcriptome analysis for R and H under salt stress. (**A**) the biplot of PC1 and PC2 for up-regulated DEMs. (**B**) Heatmap for hierarchical clustering of 12 samples from R and H. (**C**) Volcano plot representing the regulation pattern of DEMs. (**D**) Statistics of up-regulated and down-regulated DEMs in roots. (**E**) The KEGG enrichment analysis of up-regulated DEMs in R4 vs H4. Vertical columns represent enriched pathways and horizontal columns are for annotated metabolites. (**F**) Integration of transcriptome and metabolome data. Green color represents a particular metabolite, orange color represents the enzyme catalyzing a particular reaction, and yellow color stands for the production or consumption of energy in a chemical reaction. ALDH, aldehyde dehydrogenase; CoA, coenzyme A; DEM, differentially expressed metabolites; H, *A. deliciosa*; LDH, L-lactate dehydrogenase; PC, principal component; PDC, pyruvate decarboxylase; PDH, pyruvate dehydrogenase; PK, pyruvate kinase; R, *Actinidia valvata*.

Based on GO term results of metabolic processes enrichment, the widely targeted metabolic profiles for R and H were performed under salt stress. Three repetitions for each group were implied to enhance the reliability of experimental data and results. The PCA analysis separated metabolites into two distinct areas of the plot (Fig. 4A). Similarly, the hierarchical clustering for samples resulted in two distinct clades for both species (Fig. 4B). Further analysis revealed that 70 metabolites were up-regulated and 71 metabolites were down-regulated in R4 vs H4 under salt stress (Fig. 4C and 4D upper panel). The Venn diagram results showed the presence of 37 differentially expressed metabolites in R4 vs H4 (Fig. 4D, lower panel).

### Differentially expressed metabolites under salt stress

The KEGG pathways analysis for the top 20 metabolic pathways revealed several salt stress-responsive pathways which include flavonoid biosynthesis, pyruvate metabolism, phenylpropanoid biosynthesis, glutathione metabolism, etc. (Fig. 4E). For the identification of key pathways involved in salt stress tolerance, we compared the KEGG pathways for transcriptome and KEGG pathways for metabolome. This comparison resulted in the identification of three overlapping pathways, namely pyruvate metabolism, phenylpropanoid biosynthesis, and glutathione metabolism. These overlapping pathways might play important role in enhanced salt stress tolerance of R.

### Integration of transcriptome and metabolome data reveals pyruvate biosynthesis as an important source of energy for R against salt stress

Based on the statistically significant *q*-_value_ of KEGG pathways and overlapping with RNA-seq KEGG results, we selected the pyruvate metabolism pathway for integration of transcriptome and metabolome analysis results (Figs 4E, 1Cii). The results of pyruvate contents showed its enhanced accumulation under an extended time course of salt stress in kiwifruit (Fig. 2Diii). To determine the association between the pyruvate biosynthesis pathway and its associated genes, we screened out the differentiability expressed genes from R4 vs H4 involved in each step of this pathway. We identified 10 DEGs for *enolase* among which R_transcript_40556 had a significantly higher expression, 19 genes for *pyruvate kinase* (*PK*) among which R_transcript_15014 showed higher expression, three genes for *L-lactate dehydrogenase* (*LDH*) and H_transcript_93191 showed higher expression among all, nine genes for *pyruvate decarboxylase* (*PDC*) in which R_transcript_13128 showed differential expression, 35 genes for *aldehyde dehydrogenase* (*ALDH*) among which R_transcript_9286 gene showed significantly different expression in R. Similarly, there were 10 genes for acetyl-coenzyme A synthetase (*CoA*) among which R_transcript_48519 was differentially expressed between both species, while 40 genes were found for *pyruvate dehydrogenase* (*PDH*) in which the R_transcript_58755 gene showed significant changes in expression for R (Fig. 4F).

### Functional validation of *AvBADH* in *at* plants

**Figure 5:**
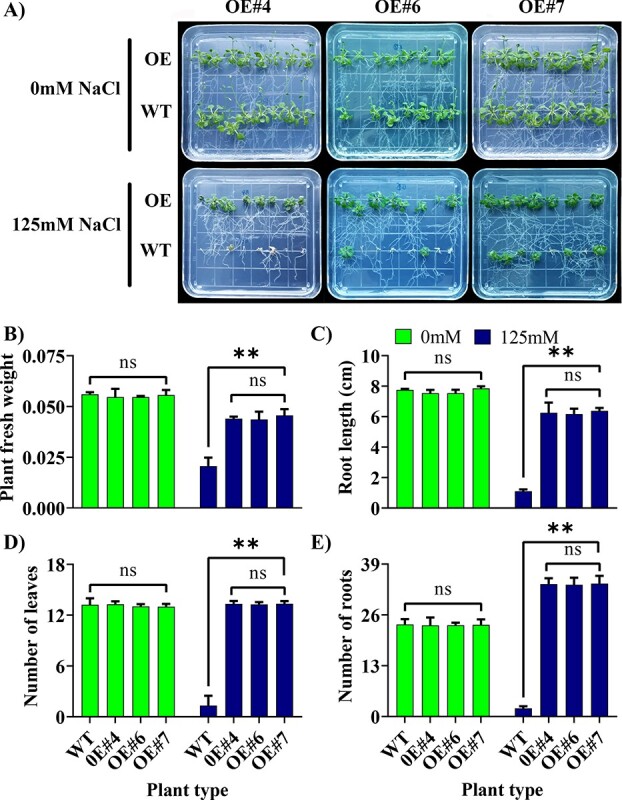
Functional validation of *AvBADH* gene in early-stage *At* plants under 300 mM NaCl treatment. **(A**) Phenotype for early-stage transgenic *At* plants. (**B**) Plant fresh weight (g), (**C**) root length (cm), (**D**) number of leaves, (**E**) number of roots. Each value represents the mean ± SD of three biological replicates. The ns represents non-significance mean differences while ** denotes significant mean differences at *P* ≤ 0.01. ns, non-significant; OE, overexpression; WT, wild type.

**Figure 6:**
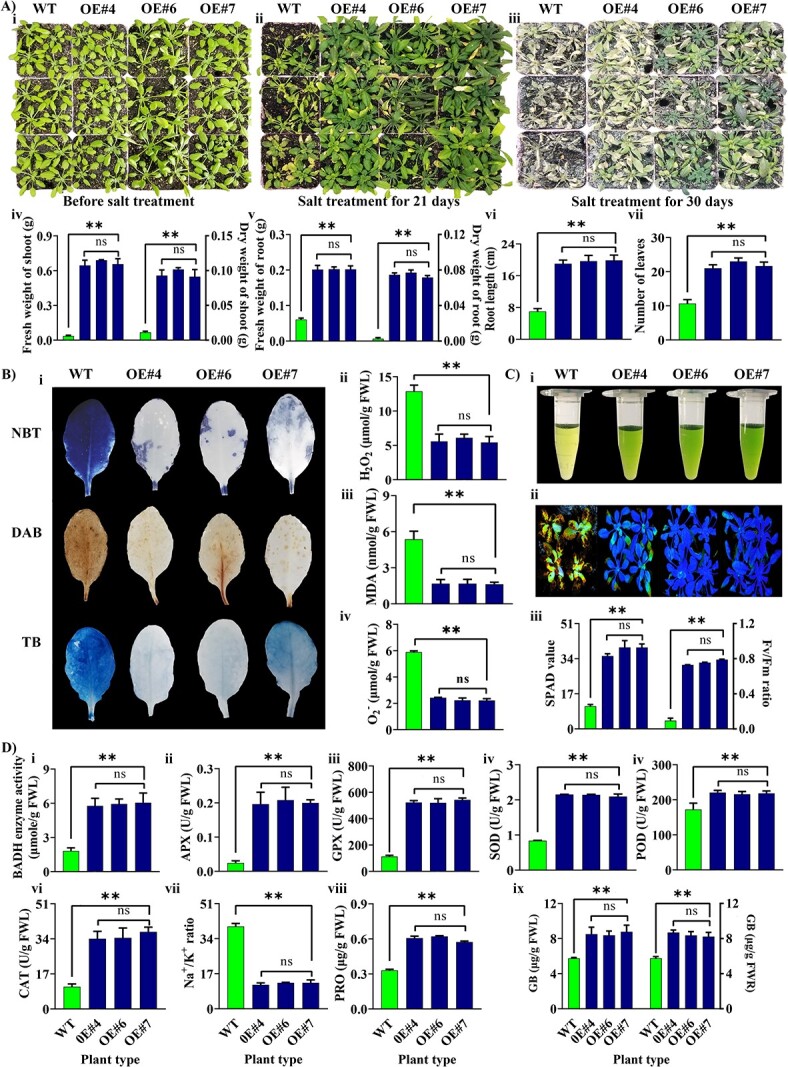
Functional validation of *AvBADH* gene in late-stage *At* plants under 300 mM NaCl treatment. (**A)** Phenotypes for transgenic lines and WT *A. thaliana*. (**i**) transgenic and WT lines before salt stress treatment, (**ii**) transgenic and WT lines after 300 mM salt stress for 21 days, (**iii**) transgenic and WT lines after 300 mM salt stress for 30 days, (**iv**) fresh and dry weight of shoot (g), (**v**) fresh and dry weight of root (g), (**vi**) root length (cm), and (**vii**) the number of leaves. (**B)** Histochemical staining of transgenic and WT lines under 300 mM salt stress for 21 days. (**i**) NBT (first row), DAB (second row), and Trypan blue (third row), (**ii**) accumulation of hydrogen peroxide (H_2_O_2_), (**iii**) accumulation of malonaldehyde (MDA), and (**iv**) accumulation of oxygen radicle (O_2_^−^). (**C**) Chlorophyll analysis for transgenic and WT lines under 300 mM salt stress for 21 days. (**i**) Extracted chlorophyll from leaves, (**ii**) chlorophyll fluorescence, (**iii**) SPAD value and Fv/Fm ratio. (**D**) Assays of (**i**) BADH enzymes, (**ii**) APX, (**iii**) GPX, (**iv**) SOD, (**v**) POD, (**vi**) CAT, (**vii**) Na^+^/K^+^ ratio, (**viii**) PRO, and (**ix**) GB. Each value represents the mean ± SD of three biological replicates. The ns represents non-significance mean differences while ** denotes significant mean differences at *P* ≤ 0.01. ns, non-significant; OE, overexpression; WT, wild type.

The glycine betaine pathway was deliberately selected due to the two-step conversion of choline (a toxic substance) to betaine (a harmless substance) in the presence of *CMO* and *BADH* genes. The second step in this pathway is rate liming. The phylogenetic analysis results showed that *AvBADH* was clustered with *Avicennia marina* (Fig. S6, see online supplementary material). The heatmap for 13 *AvBADH* genes from transcriptome data is given in supplementary files (Fig. S6, see online supplementary material). The sub-cellular localization results suggested that *AvBADH* gene was localized in peroxysomes and chloroplast of tobacco plant cells (Fig. S6, see online supplementary material). The successful T_1_ transformants were detected by RT-PCR (Fig. S6, see online supplementary material). We carried out a germination test and pot experiment to assess the performance of T_3_ generation transgenic lines and WT plants under salt stress. The early-stage and late-stage *At* plants were treated with 125 mM salt or no salt, and 300 mM salt, respectively. The roots and shoots of transgenic *At* plants showed higher relative expression of *AvBADH* gene than WT plants (Fig. S6, see online supplementary material). Under normal conditions, the *At* seedlings overexpressing *AvBADH* and WT had similar growth (Figs 5A and 6Ai). In contrast transgenic plants performed better than WT plants under salt stress (Figs 5A and 6Aii, iii). Salt stress negatively impacted growth parameters in WT and overexpressed lines. However, the growth parameters (number of leaves, root length, fresh and dry weight of shoot and root, plant fresh weight, germination and survival rate) of early-stage and late-stage *At* seedlings overexpressing the *AvBADH* gene were significantly higher than WT plants (Figs 5B, C, D, 6Aiv, v, vi, vii, and Fig. S6, see online supplementary material).

**Figure 7:**
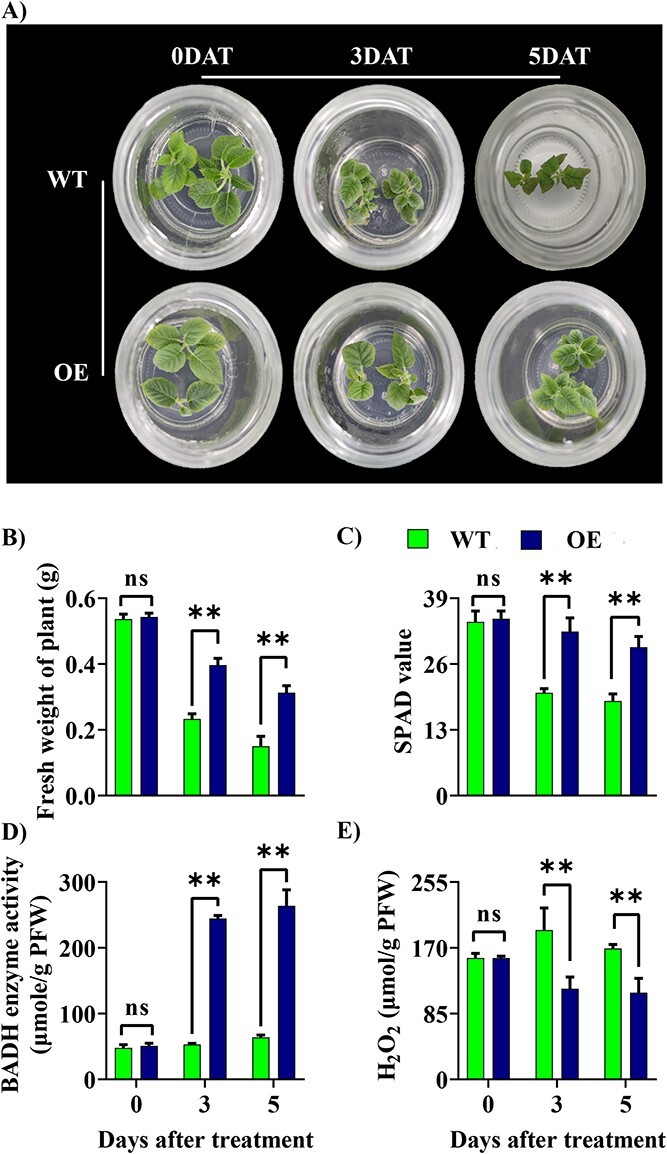
Functional validation of *AvBADH* gene in kiwifruit under 0.4% salt stress for 0 days, 3 days, and 5 days. (**A**) Phenotype for transgenic and WT lines. (**B**) Fresh weight (g) of plants. (**C**) Chlorophyll contents (SPAD value). (**D**) BADH enzyme activity. (**E**) Accumulation of H_2_O_2_. Each value represents the mean ± SD of three biological replicates. The ns represents non-significance mean differences while ^**^ denotes significant mean differences at *P* ≤ 0.01. ns, non-significant; OE, over expression; WT, wild type.

The histochemical staining results showed significantly lower production of H_2_O_2_, O_2_^−^, and MDA in transgenic lines over-expressing *AvBADH* than WT under 300 mM salt stress for 21 days (Fig. 6B). The *At* plants over-expressing *AvBADH* produced significantly higher chlorophyll contents and Fv/Fm compared to WT plants (Fig. 6C). The transgenic lines over-expressing *AvBADH* showed higher BADH enzyme activity and accumulated higher Na^+^, K^+^, Cl*^−^*, Na^+^/K^+^ ratio, APX, GPX, CAT, Proline, and glycine betaine than WT plants under 300 mM salt stress for 21 days (Fig. 6D and Fig. S12, see online supplementary material).

### Functional validation of *AvBADH* in *Ac* plants

We constructed an overexpression vector for the *AvBADH* gene to perform the transformation in *Ac* (‘Hongyang’) through *Agrobacterium*-mediated transformation. The positive transgenic lines were detected by using specific pair of primers through RT-PCR (Fig. S13, see online supplementary material). The WT and transgenic plants were grown on an MS medium containing 0.4% (w/v) salt (NaCl). The plant samples were taken at zero-day (control), three days, and five days after salt stress treatment for subsequent analysis. The transgenic plants showed higher relative expression of *AvBADH* gene than WT plants (Fig. S14, see online supplementary material). The phenotype analysis showed that transgenic lines performed better than WT plants after five days of salt stress (Fig. 7A). The transgenic plants showed improved fresh weight, chlorophyll contents, and BADH enzyme activity than WT plants under salt stress (Fig. 7B, C, and D). On the contrary, the transgenic lines accumulated less H_2_O_2_ than WT plants under salt stress, suggesting that overexpression of the *AvBADH* gene significantly improved ROS scavenging in transgenic kiwifruit plants (Fig. 7E).

## Discussion

Kiwifruit is a recently domesticated and relatively new fruit plant. It is highly popular among consumers due to its remarkably high vitamin C content [27]. Unfortunately, it is highly prone to abiotic stress especially salt stress because of its fleshy and shallow root system. Therefore, the kiwifruit industry is under constant pressure to develop new rootstocks that can assist plants to thrive well in salt-affected areas. In the past, the seedlings from H species have widely been used as rootstock which is sensitive to environmental stresses. Recently, the R species showed high potential for rootstock as ZMH genotype performed very well against different abiotic stress [29, 43]. Keeping in view the above-mentioned results, the integrated transcriptome and metabolome study was designed to underpin the fundamental difference in regulatory pathways between both species under salt stress.

### The ABA signal transduction is involved in salt stress tolerance

Plant hormones are signaling molecules that are reported to be actively involved in salt stress tolerance [44–48]. The salt-responsive hormones contain ABA (abscisic acid), JA (jasmonic acid), and SA (salicylic acid) [49]. ABA is the most important salt-responsive phytohormone that, under high levels, activates kinase cascades and regulates gene expression in response to salt stress. Also, increased endogenous levels of ABA under salt stress can regulate osmotic stress by closing stomata [50]. The SnRK2s (sucrose non-fermenting-1 related protein kinase 2) play an important role in osmoregulation under salt stress. In the absence of ABA, the PP2Cs (protein phosphatase 2Cs) inhibit the kinase activities of SnRK2s. Upon osmotic stress, the ABA receptor proteins including PYL (PYR1-like) and RCAR (regulatory component of ABA receptor) can inhibit the activity of PP2Cs by perceiving and binding to accumulated ABA, thus restoring the activity of SnRK2s [51]. In the present study, we observed a higher expression level of R_Transcript_19927 (abscisic acid receptor PYL9) and ABA contents in kiwifruit under salt stress (Fig. 9B, C), indicating that the ABA signaling pathway played an important role in salt tolerance of R by mitigating osmotic stress. Our results were on a par with previous studies where increased ABA gene expression [52] and elevated ABA contents [53] were reported under salt stress.

### Glycine betaine and TSS play role in salt stress tolerance

Glycine betaine is one of the major osmoprotectants along with TSS that accumulate in plants under salt stress to protect photosynthetic machinery, the structural integrity of proteins, enzymes, membranes, and scavenge ROS [54]. The soluble sugars play an important role in nutrition, osmoprotection and act as metabolite signaling molecule under salt stress in plants [55]. The overaccumulation of soluble sugars serve as carbon source and osmoprotection, maintain osmotic homeostasis and scavenge ROS to cope with the detrimental effects of salt stress in plants [56]. GB can reduce potassium efflux [57], regulate phosphate homeostasis, and increase the enzymatic activity of H^+^-ATPase in plasma membrane under salt stress [58], indicating that GB influences the regulation of ion homeostasis in plant organs. The GB is synthesized by two-step oxidation of choline via choline monooxygenase (CMO; EC 1.14.15.7) and betaine aldehyde dehydrogenase (BADH; EC 1.2.1.8) [59]. The multifunctional *BADH* gene responsible for the second oxidation reaction in the GB biosynthesis pathway is reported for salt stress tolerance, production of fragrance, and selectable marker for transgenic plants [60]. In the present study, the higher expression levels of the *AvBADH* gene and higher contents of GB and TSS protected plants from ionic and oxidative stress by maintaining ion homeostasis and providing osmo-protection, respectively.

### Glutathione metabolism helps in scavenging ROS under salt stress.

Enzymatic and non-enzymatic antioxidants involved in ROS scavenging are key players in alleviating oxidative damage in plants caused by salt [61]. Glutathione is a non-enzymatic antioxidant that is found widely in plant tissues. During ROS scavenging, the glutathione is converted to GSSG (glutathione disulfide) by GPX (glutathione peroxidase) enzyme, while GSSG is recycled again to glutathione by GR (glutathione reductase) enzyme. The GPX and GR enzymes are responsible for maintaining glutathione homeostasis and glutathione/GSSG ratio [62]. In the plant cell, the glutathione reductase (GR) gene is responsible for maintaining the supply of reduced glutathione under salt stress. Therefore, the higher expression level of GR and higher Glutathione in salt-tolerant kiwifruit suggested that glutathione played an important role in ROS savaging and maintaining ion homeostasis under salt stress.

### Pyruvate metabolism acts as an energy source for kiwifruit during salt stress

Respiration is an important metabolic process that is present in almost all living organisms on the surface of the earth. The key role of pyruvate, an end product of glycolysis, in plants is to provide energy in the form of ATP [63]. Additionally, pyruvate enters into chloroplast through transporter genes (i.e. *BASS2*) and serves as a precursor for ABA signaling. During glycolysis, phosphoenol pyruvate is converted to pyruvate in the presence of the pyruvate kinase (PK) enzyme. The pyruvate kinase enzyme needs K^+^ ion in plants as a co-factor for its activity under salt stress. Previously, pyruvate showed decreased expression on overconsumption of proline in the Krebs cycle in *A. lagopoides* [64]. Thus, we inferred that pyruvate not only provided energy to plant cells but in the presence of the K^+^ ion served as a precursor for ABA signaling to confer salt tolerance in kiwifruit plants.

### Mechanisms underlying the enhanced salt tolerance in R

**Figure 8:**
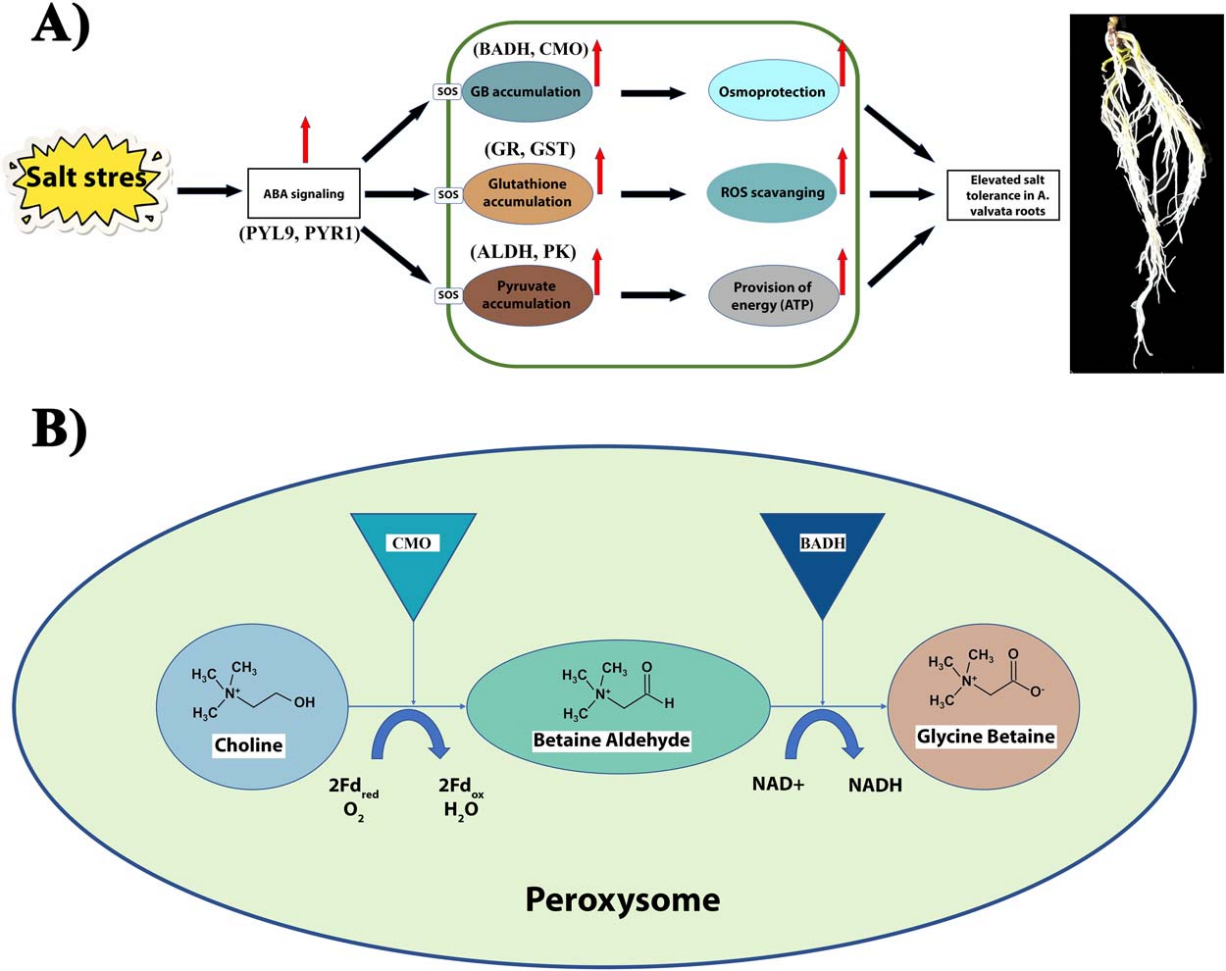
Proposed model for **(A**) molecular mechanisms underlying enhanced salt tolerance in R*,* and (**B**) The biosynthetic pathway of GB in plants. ABA, abscisic acid; BADH, betaine aldehyde dehydrogenase; CMO, choline monooxygenase; GB, glycine betaine; GR, glutathione reductase; GST, glutathione-s-transferase; ROS, radioactive oxygen species.

The present study revealed the enhanced salt tolerance of R compared to H. The results from transcriptome and metabolome analysis enabled us to propose a model to explain the potential mechanism underlying the enhanced salt tolerance in R. Upon exposure to salt stress, the R roots underwent the activation of the ABA signaling pathway. The plant cells received these signals and activated the biosynthesis of metabolites involved in salt stress tolerance. The overaccumulation of GB and TSS protected plants against osmotic stress. Similarly, the glutathione biosynthesis safeguarded plants from ionic stress through scavenging ROS. On the other hand, over-accumulation of pyruvate biosynthesis provided an adequate amount of energy in the form of ATP to carry out uninterrupted cell functioning (Fig. 8A). During the biosynthesis of GB, choline (a toxic substance) is oxidized into glycine betaine (an osmo-protectant) in the presence of CMO and BADH enzymes (Fig. 8B).

## Conclusion

In the present study, we comprehensively analysed the global changes in transcriptome and metabolome profiles in two kiwifruit species with contrasting salt tolerance abilities. The KEGG pathway enrichment analysis identified key pathways that are directly involved in salt stress tolerance. The WGCNA analysis identified two co-expression modules that were highly correlated with changes in ABA, glutathione, GB, TSS, and pyruvate contents. Furthermore, five candidates that were weighted as candidate hub genes were identified within these modules. Our results suggested that salt stress tolerance in kiwifruit could not be attributed to a single gene or metabolite, but to an intricate regulatory mechanism of pathways. Additionally, the *AvBADH* gene from GB pathway enhanced salt tolerance of transgenic *A. thaliana* and *A. chinensis* plants. Future research on salt stress tolerance should be directed to the candidate genes and metabolic pathways identified in this study.

## Materials and methods

### Plant material and salt treatment

We selected two *Actinidia* species *A. valvata* Dunn. (ZMH, tolerant) and *A. deliciosa* Planch. var. *deliciosa* (cultivar ‘Hayward’, HWD, sensitive) based on our previous genotype screening study for salt stress [29]. Plants for both genotypes were propagated through the tissue culture technique on Murashige and Skoog (MS) medium [65] containing 30 g/L sucrose and 7.2 g/L agar (pH 5.8) to obtain true-to-type plants. The explants for both genotypes were obtained from Zhengzhou Fruit Research Institute, Chinese Academy of Agricultural Sciences, China (latitude 34°43′N, longitude 113°39′E, and altitude 111 m). After rooting, plantlets were transferred into 6 × 6 cm plastic pots with a 3 L holding capacity containing growing media (compost: perlite: vermiculite = 2:1:1). At the 4–6 leaf stage, plants were subjected to 0.4% NaCl per net weight of the growing medium in the pot. Treatment was repeated thrice and there were five plants in each replication. Samples from plant roots were collected at 12 h, 24 h, and 72 h after treatment, considering 0 h as control.

### Physiological measurements

The pyruvate contents were measured according to a previously described method [66]. The ABA (abscisic acid) contents were measured by following a previously described protocol [67]. The GB (glycine betaine) and TSS (total soluble sugar) were measured by using commercial kits (Nanjing Jiancheng Bioengineering Institute, Nanjing, China), while the glutathione was measured according to the previously described protocol [68]. A bio-mate 3 s spectrophotometer (Thermo Scientific, Waltham, MA, USA) was used to check the optical density (OD).

### RNA extraction and RNA sequencing

Samples for RNA-seq analysis were collected from the roots of two contrasting kiwifruit genotypes for salt stress. In total, 24 samples were collected from the control and three time-points for both genotypes (2 cultivars × 4 sample collection times × 3 biological replicates × 5 technical replicates). All the samples were sent to Biomarker Technology Company (Biomarker, Beijing, China) for RNA-seq. Total RNA from the roots was isolated according to the manufacturer’s protocol of the Plant Total RNA purification kit (Waryoung, Beijing, China). We used NanoDrop 2000 (Thermo Scientific, MA, USA) system and the NanoDrop 6000 assay kit (Agilent technologies, Beijing, China) to check the RNA concentration and integrity. The cDNA libraries were synthesized from RNA by using magnetic beads linked with Oligo (dT) to enrich eukaryotic mRNA and their concentration was assessed by Agilent Bioanalyzer 2100 system (Agilent Technologies, CA, USA). RNA-seq libraries were sequenced by an Illumina Hiseq platform X Ten (Illumina, CA, USA). To construct PacBio libraries, cDNA was synthesized by using SMRTer PCR cDNA synthesis kit (Clontech, USA) and the PacBio libraries were constructed according to the PacBio protocol (http://www.pacb.com/wp-content, accessed on November 15, 2020).

### RNA-seq Data processing

Paired-end raw reads were filtered by in-house perl script to remove adapters, primer sequences, low-quality reads, and reads containing poly (N) to obtain the clean reads in FASTQ format. At the same time, a base distribution map was used to calculate the Q20, Q30 AT and GC contents, and sequence duplication level of clean reads. The SMART Analysis Software suit was used to analyzed the sequencing results from PacBio (http://www.pacb.com/product-and-services /analytical-software/smrt-analysis, accessed on November 15, 2020). The SMART sequencing results were filterd for high quality reads by removing reads with a quality of < 0.75 and length of < 50bp. The high-quality reads were then used to obtain error-corrected circular consensus sequences (CCSs) with the full passes = 0 and quality > 0.75. The CCSs were considered as full-length (FL) transcripts only if the 5′-primer sequence, 3′ primer sequence and a poly A tail were present. The nonchimeric sequences in the FL sequences were considered to be FL-nonchimeric (FLNC) sequences. The FLNC sequences with high-quality bases were obtained for subsequent analysis.

### Functional annotation of transcripts and identification of DEGs

The reference transcriptome was assembled de novo from SMART transcript data due to unavailability of reference genome for hexaploid kiwifruit. The clean reads from RNA-seq data were then mapped to the Pacbio reference genome sequence by using Hisat2 tool [69]. The reads with perfect match or one mismatch were used for subsequent analysis. The uni-gene sequences were annotated against KEGG (Kyoto Encyclopedia of Genes and Genomes), GO (gene ontology), NR (non-redundant protein sequence), Pfam (homologous protein family), KOG (eukaryotic ortholog group), and Swiss-Port databases to obtain their annotation by using local blast programs at a threshold E-value = 1e-5. The plant transcription factors (TFs) and TF family classification was predicted by iTAK [70]. The expression levels of the gene were calculated by fragment per kilobase of transcript per million fragments mapped (FPKM) method. Identification of DEGs (Differentially expressed genes) was done by DESeq2 [71] and edgeR R package [72]. The genes were considered differentially expressed at FDR-adjusted P-value < 0.01 and |log2 (fold change) | 2. The enriched DEGs via GO (gene ontology) [73] and KEGG (Kyoto Encyclopedia of Genes and Genomes [74] were used to identify significant metabolic pathways at an FDR-adjusted P-value < 0.05.

### qPCR validation of selected DEGs

For the expression validation, 16 DEGs were randomly selected from RNA-seq data to analyse the relative gene expression levels [75]. Total RNA from the roots was isolated according to the manufacturer’s protocol of the Plant Total RNA purification kit (Waryoung, Beijing, China). The RT-qPCR was performed according to a previously described method [76]. All the experiments were repeated thrice. The relative expression of genes was calculated by 2^ΔΔCT^ method [77]. All the pair of primers used for RT-qPCR are listed in Table S1 (see online supplementary material).

### WGCNA for identification of regulatory networks and hub genes

We performed WGCNA by using a R-based WGCNA package (v.1.69) with default parameters [78]. The adjacency matrix was constructed by using the FPKM values of DEGs, which was then converted to TOM (topological overlap matrix). Modules were detected by using a dynamic hybrid tree-cut algorithm (DynamicTreeCut, v.1.63–1) with default parameters [79]. The color modules were identified based on phenotype data, and the correlation matrix between phenotype and gene module was computed by an R-based WGCNA package. The networks for color modules were visualized by Cytoscape software (v.3.8.2) [80].

### Metabolomic profiling and statistical analysis

The extraction of metabolites from R roots was performed according to the previously described protocol with slight modifications [81]. Primary metabolites were quantified through GC–MS measurements and LC–MS was implied to measure secondary metabolites. Annotation for LC–MS data was done by matching the extracted data from the chromatogram KEGG compound database and KEGG pathway database. An R-based package TargetSearch was used to annotate GC–MS data [82]. The normalized data were subjected to PCA analysis on R-based software Factoextra (v.1.0.6) with default parameters [83]. The Metaboanalyst (v.4.0) software was used to choose DEMs [84]. The VANTED software was used to construct metabolic pathways [85].

### Gene cloning, phylogenetic tree, and subcellular localization analysis

For stable transformation in *At* and *Ac*, the open reading frame (ORF) of *AvBADH* was cloned from cDNA of R roots by using a specific pair of primers (Table S4, see online supplementary material). The phylogenetic tree for BADH proteins from R and other plant species was constructed by GenomeNet software (https://www.genome.jp/) and visualized by iTOOLs software. For subcellular localization analysis, the coding sequences (CDSs) of *AvBADH* was amplified by using a specific pair of primers lacking stop codon (TAG). The PCR product was transferred into a pBI121-GFP vector driven by CaMV 35S promoter. After sequence confirmation, the recombinant 35S pro:*AvBADH*::GFP, and 35S pro:GFP (control) plasmids were entered into an *Agrobacterium tumefaciens* strain GV 3101. The cultures of recombinant plasmids in GV3101 (OD_600_ = 0.6–1.0) were injected into tobacco plant leaves and kept in a growth chamber for 2–3 days. The transformed leaves were analysed on a confocal laser-guided microscope (FV1000; Olympus, Tokyo, Japan) for signal detection.

### Transformation of *At* and *Ac* plants

For *At* transformation, the ORF of *AvBADH* was cloned into pBI121 vector driven by CaMV 35S promoter. *A. tumefaciens* strain GV3101 harboring the *AvBADH* was transformed into WT *At* (Columbia-0) via floral dip method [86]. The selection of T_0_ positive seeds was done on MS medium containing kanamycin (100 mg/L) (Sigma, Saint Louis, MO, USA). The detection of positive plants was done by RT-qPCR with F-35S + R-BADH primer pair. The positive plants were raised to T_3_ generation to ensure the maximum homozygosity. Three positive T_3_ transgenic lines were selected for subsequent functional validation.

For *Ac* transformation, the ORF of *AvBADH* was cloned into pBI121 vector driven by CaMV 35S promoter. The *A. tumefaciens* strain EHA105 harboring the *AvBADH* was transformed into *Ac* leaves by following a previously described protocol [87]. We obtained transgenic plants after ~6 months. The positive transformants were identified by PCR and RT-qPCR analysis. Three positive transformants with higher expression levels were used for subsequent analysis.

### Assay of salt stress for transgenic *At* plants

#### Early-stage *At* plants

For germination assay, we germinated eight seeds per plate for WT and each OE line on MS medium plates containing 0 mM or 125 mM NaCl salt concentration. Each treatment was replicated on three plates. The germination rate was calculated seven days after transferring plates into the light. The germination was confirmed on the emergence of plumule from seed coat [88]. The Root length was measured in centimeters (cm). The fresh weight of root and shoot was measured in grams (g) on a weight balance immediately after taking samples. The shoot and root samples were oven-dried at 65°C for 72 hours for taking dry weight in grams (g). Plant survival rate was measured by taking the percentage (%) of alive plants for each line.

#### Late-stage *At* plants

The 15-day-old transgenic and WT *At* potted plants were treated with 300 mM NaCl salt stress. For measuring physiochemical parameters, samples were taken from plants after 21 days of no-salt or 300 mM NaCl salt stress treatments. The total chlorophyll contents were measured according to a previously described protocol [89]. The ion contents for Na^+^, K^+^, and Cl^−^ were measured following previous methods [90, 91]. The contents for APX (Ascorbate peroxidase, EC 1.11.1.11) GPX (Glutathione-s-transferase, EC 2.5.1.18), CAT (Catalase, EC 1.11.1.6), POD (Peroxidase, EC 1.11.1.7) and SOD (Superoxide dismutase, EC 1.15.1.1) were measured according to previously described methods [90, 92, 93]. BADH enzyme activity was carried out by enzyme-linked immunosorbent (ELISA) assay method using a specific detection kit (ml036337 for BADH activity, Mlbio, Shanghai, China) and membrane damage (MDA) was measured according to the method described by Chen and his team [94]. Proline and GB were measured by previously described methods [95]. A fluorimeter (IMAGING-PAM) was used to record chlorophyll fluorescence and Fv/Fm was calculated by Imaging WinGegE software. The histochemical staining for O_2_^.--^, H_2_O_2_, and plant cell death was done with nitro blue tetrazolium (NBT), 3,3′-diaminobenzidine (DAB), and trypan blue, respectively [96, 97]. The H_2_O_2_ and O_2_^.--^ were measured calorimetrically [98, 99].

##### Transcriptional activation activity (TA) analysis of *AvMYB* TF

The transcriptional activation (TA) activity was carried out by following the steps described in a previous study [100]. The coding sequence (CDS) of *AvMYB* was amplified from cDNA of R and inserted into pGBKT7 vector using Novoprotein recombination kit to form pGBKT7-*AvMYB1* and pGBKT7-*AvMYB2* vectors (Novoprotein, Suzhou, China). Then, the constructs and empty pGBKT7 vector (negative control) were transferred into AH109 yeast strain. Transformed colonies were screened on SD/−Trp, SD/−Trp-His-Ade, and SD/−Trp-His-Ade with X-a-Gal solid media, which were cultured 2–3 days in an incubator at 30°C.

### Statistical analysis

Physiological data were analysed for significant differences by one-way ANOVA and *t-test*. The mean differences were computed through Duncan’s MRT test. The results were presented as means ± SD. All the experients were repreated thrice and all the analyses were carried out with Mac-based R packages.

## Core idea

- To the best of our knowledge, this is the first-ever study on kiwifruit to decipher intricate regulatory mechanisms involved in salt tolerance through integrated transcriptome and metabolome analysis.
- The hexaploid R (ZMH) is a special plant material that offers enhanced salt tolerance.
- Gene co-expression network analysis resulted in the identification of two novel genes pyruvate kinase (R_transcript_13859) and fructokinase (H_transcript_39638) related to pyruvate and sugar metabolism in R under salt stress.
- The pyruvate metabolism is rarely reported to be involved in salt stress studies. In the present study, the pyruvate metabolism was also identified among important pathways contributing to elevated salt tolerance in R plants under salt stress.
- The *At* and *Ac* plants overexpressing the *AvBADH* gene exhibited elevated salt tolerance compared to WT plants under different salt stress concentrations.

## Supplementary Material

supp_data_uhac189

## Data Availability

The authors declare that the data supporting the findings of this study are presented in the article and supplementary information files are available from the corresponding author upon request. Transcriptome raw data is submitted in NCBI public repository under BioProject (PRJNA726156).
