# Supplementary material for: Comparative transcriptome and metabolome analysis reveal key regulatory defense networks and genes involved in enhanced salt tolerance of Actinidia (kiwifruit)
Source: Hortic Res. 2022 Aug 25;9:uhac189. doi: 10.1093/hr/uhac189 (PMC9630968; doi:10.1093/hr/uhac189)
Supplement: supp_data_uhac189 [file supp_data_uhac189.zip › Supplementary material.zip!/Supplementary material.docx]

**Supporting information**

**Title: Comparative transcriptome and metabolome analysis reveal key regulatory defense networks and genes involved in enhanced salt tolerance of *Actinidia* (kiwifruit)**

**Author names and affiliations:** Muhammad Abid^1,2,†^, Shichao Gu^1,†^, Yong-Jie Zhang^1^, Shihang Sun^1^, Zhi Li^1^, Dan-Feng Bai ^1^, Liming Sun^1^, Xiu-Juan Qi^1^, Yun-Peng Zhong^1,*^, Jin-Bao Fang^1,*^

^1^Key Laboratory for Fruit Tree Growth, Development and Quality Control, Zhengzhou Fruit Research Institute; Chinese Academy of Agricultural Sciences, Zhengzhou 450009 China.

^2^ Lushan Botanical Garden, Chinese Academy of Sciences, Jiujiang 332900, P.R. China

**Fig. S1. Venn diagrams for DEGs in different comparison pairs**. **A)** Comparison of DEGs in *Av* and *Ad* under 0h and 12h, **B)** Comparison of DEGs in *Av* and *Ad* under 0h and 24h, and **C)** Comparison of DEGs in *Av* and *Ad* under 0h and 72h.

Fig. S2. Expression profile of 16 randomly selected DEGs from RNA-seq data through RT-qPCR analysis. Please rotate figure anti-clockwise to read.

Number of DEGs

| Names of plant harmones | Up-regulated DEGs |
| --- | --- |
| Auxin | 176 |
| ABA | 112 |
| Cytokinin | 113 |
| BR | 110 |
| Ethyline | 106 |
| GA | 39 |
| JA | 77 |
| SA | 100 |

**Fig. S3.** Number of up-regulated DEGs in each plant hormone signaling family in *Av* and *Ad* under salt stress.

**Fig. S4.** The clustered dendrogram of DEGs from *Av* and *Ad* under salt stress.

Total Soluble Sugars (mg/g FW)

| Time course for 0.4% NaCl treatment | ZM-H | HWD |
| --- | --- | --- |
| 0h | 99.3211 | 99.7832 |
| 12h | 98.7169 | 65.2342 |
| 24h | 74.5468 | 48.1019 |
| 72h | 69.3574 | 31.3962 |

**Fig. S5.** Changes in total soluble sugars (TSS) in *Av* and *Ad* under salt stress. Each value represents the mean ± SD of three biological replicates.

**Fig. S6.** Phylogenetic tree for BADH proteins *Av* and other plant species.

**Fig. S7.** Heatmap analysis of 13 *AvBADH* genes in *Av* and *Ad* under 0.4% NaCl stress.

**Figure S8. Subcellular localization of *AvBADH* gene in tobacco plants.**

**Fig. S9. RT-PCR detection of T_1_ transgenic** *At* **plants**. M stands for 2000 bp marker, - stands for ddH2O sample, + stands for recombinant plasmid containing *AvBADH* gene, WT stands for wildtype kiwifruit, and number 1-12 stands for transgenic lines. The forward primer was designed at 5’ of 35S and reverse primer was designed at 3’ of *AvBADH* gene.

**Fig. S10.** Relative expression of *AvBADH* gene by RT-qPCR in roots and leaves of late-stage *At* plants under 300 mM salt stress for 21 days.

**Fig. S11. Analysis of** A) Germination test of early-stage *At* plants, and B) Survival rate of late-stage *At* plants under 300 mM salt stress for 21 days.

**Fig. S12. Assays for** A) Na^+^ (sodium) ion, B) K^+^ (potassium) ion, and C) Cl^-^ (chloride) ions in late-stage *At* plants under 300 mM salt stress for 21 days.

**Fig. S13. RT-PCR detection of T_1_ transgenic** *Ac* **plants**. M stands for 2000 bp marker, - stands for ddH2O sample, + stands for recombinant plasmid containing *AvBADH* gene, WT stands for wildtype kiwifruit, and number 1-6 stands for transgenic lines. The forward primer was designed at 5’ of 35S and reverse primer was designed at 3’ of *AvBADH* gene.

**Fig. S14.** Relative expression of *AvBADH* gene by RT-qPCR in *Ac* plants under 0.4% (w/v) salt stress.

**Table S1.** Summary of data for PacBio RSII and Illumina Hiseq

| **Platform** |  |
| --- | --- |
| **PacBio RSII** |  |
| Total CCS for H | 427,703 |
| Total CSS for R | 278,183 |
| FLNC reads for H | 374,941 |
| FLNC reads for R | 175,112 |
| FLNC percentage for H | 87.66% |
| FLNC percentage for R | 62.95% |
| Total unignes for H | 103,582 |
| Total unignes for R | 77,398 |
| TF number | 20,597 |
| **Illumina Hiseq** |  |
| Clean reads (Average) | 21,973,249 |
| Clean base (G) | 6,555,153,763 |
| Q20 (average) | 98.107% |
| Q30 (average) | 94.394% |
| GC contents (Average) | 46.797 |
| PC1 | 25.63% |
| PC2 | 13.68% |
| PC3 | 12.33% |

**Table S2.** Pairs of primers used for RT-qPCR

| **Gene ID** | **Forward Primer** | **Reverse Primer** |
| --- | --- | --- |
| **R_transcript_18876** | GGCGAACTGACAACGAGATC | TGGAGCCAGTACACTACCTG |
| **R_transcript_19574** | GGCGAACTGACAACGAGATC | CTCTTTGGTCTCCGCAACTG |
| **R_transcript_19927** | TCAAAGCCCCCGTTGATCTC | GTGCTTGTTGTGGCTGGAAG |
| **R_transcript_88504** | AACCCCAAACGTACAAGCAC | CGATTCGAGAACAACGGTCC |
| **R_transcript_92720** | GCGAGTCATATGGAGGTGGA | CCACTGCCTTGTCAACATCC |
| **H_transcript_133231** | CAATCGAGAATTAGGCGGGC | GCAAGCTCAATGGATCGGTT |
| **R_transcript_80984** | GCTGCAATACTCAAGCCCTC | TGCAACCTTGTCAACGTGAG |
| **R_transcript_45466** | AGCTCCGGTTTCTCTTCCAA | GCCCCACCTCTTTACAGACT |
| **R_transcript_68566** | GACTGAGCCCTACATCGTCA | CGTGGTTGTCGCTGTAGATG |
| **R_transcript_47811** | CGCTCCTCGTCTTCTTCTCA | AGGCCATGGTACTTGACGAA |
| **R_transcript_45295** | TGGTGTCTTTCCTGAGCACA | CACTAAGGCCAGGCTCAGTA |
| **R_transcript_55242** | TAAAGTTGGCAAAGGACGGC | TCGAAAACAGGATCCACGGA |
| **H_transcript_43672** | TAAGCAGTTGGAGGGAGAGC | GTGCTGGTCAGGAAGAGTCT |
| **R_transcript_92720** | TAGCAGCTCTCGAGACATGG | CCCGACTTTCCAAGCAAACA |
| **R_transcript_18305** | TTTGTCCAAAGAAGGCAGCG | TGATTCATCCAGTGCCAGGT |
| **H_transcript_113922** | GTGGCAAGCTTTTCGAAGGA | ACTTGACGTGCGAAGGAATG |

**Table S4.** Full names for abbreviated words

| **Sr. No.** | **Abbrivation** | **Full Name** |
| --- | --- | --- |
| 1 | 1 | 0 hours |
| 2 | 2 | 12 hours |
| 3 | 3 | 24 hours |
| 4 | 4 | 72 hours |
| 5 | ABA | Abscisic acid |
| 6 | AISAP | Aeluropus littoralis Stress associated protein |
| 7 | ALDH | Aldehyde dehydrogenase |
| 8 | AP2/ERF-ERF | Ethylene responsive factors |
| 9 | ATP | Adenosine triphosphate |
| 10 | B3-ARF | Auxin responsive factor |
| 11 | BADH | Betaine Aldehyde dehydrogenase |
| 12 | BAR1 | Adaptor protein |
| 13 | bHLH | Basic helix-loop-helix |
| 14 | BR | Brassinosteroid |
| 15 | bZIP | Basic-leucine |
| 16 | C2H2 | Cys 2 his 2 like fold group |
| 17 | C3H | Coumarate 3-hydroxylase |
| 18 | CAMK_CDPK | Calcium and calmodulin regulated kinase-Calcium dependent protein kinase |
| 19 | CCD1 | Carotenoid cleavage dioxygenases 1 |
| 20 | cDNA | Complementary deoxyribose nucleic acid |
| 21 | CoA | Co-enzyme A |
| 22 | CTK | Cytokinin |
| 23 | DEG | Differentially Expressed Genes |
| 24 | DEM | Differentially expressed metabolites |
| 25 | ERF | Ethylene responsive factors |
| 26 | FPKM | Fragments per kilobase of exon model per Million mapped fragments |
| 27 | GA | Gibberellic acid |
| 28 | GB | Glycine Betaine |
| 29 | GC-MS | Gas chromatography-mass spectrometer |
| 30 | GO | Gene ontology |
| 31 | GRAS | Gibberellic acid responsive protein |
| 32 | H | Salt sensitive (HWD) |
| 33 | h | Hours |
| 34 | HWD | Hayward |
| 35 | JA | Jasmonic acid |
| 36 | KEGG | Kyoto Encyclopedia of Genes and Genomez |
| 37 | LC-MS | Liquid chromatography-mass spectrometer |
| 38 | LDH | L-lactate dehydrogenase |
| 39 | MADS | MADX-box protein |
| 40 | MRT | Multiple Range Test |
| 41 | NaCl | Sodium Chloride |
| 42 | NHX | Na+/H+ antiporter |
| 43 | OD | Optical density |
| 44 | P5CS | Δ1-pyrroline-5-carboxylate synthetase |
| 45 | PC1 | Principle component 1 |
| 46 | PC2 | Principle component 2 |
| 47 | PCA | Principal component analysis |
| 48 | PDC | Pyruvate decarboxylase |
| 49 | PDH | Pyruvate dehydrogenase |
| 50 | PHD | Plant homeodomain |
| 51 | PK | Pyruvate kinase |
| 52 | PYL | Pyrabactin resistance 1-like |
| 53 | qPCR | Quantitative polymerase chain reaction |
| 54 | R | Salt resistant (ZM-H) |
| 55 | RLK-Pelle_DLSV | Receptor-like kinases |
| 56 | RLK-Pelle_RLCK_VIIa_2 | Receptor-like kinases |
| 57 | RLK-Pelle_XI_1 | Receptor-like kinases |
| 58 | RNA | Ribose nucleic acid |
| 59 | RNA-seq | Ribose nucleic acid sequencing |
| 60 | ROS | Radioactive oxygen species |
| 61 | SA | Sylisic acid |
| 62 | SE | Standard error |
| 63 | SET | SET domain |
| 64 | SNF | Sodium neurotransmitter symporter |
| 65 | TF | Transcription factors |
| 66 | TKL_PI-4 | Tyrosine kinase-like kinases |
| 67 | TSS | Total Soluble Sugars |
| 68 | WGCNA | Weighted gene co-expression network analysis |
| 69 | ZM-H | Zhongmei-H |

**Table S4.** Pairs of Primers used for transformation, positive transgene detection, and RT-qPCR.

| **purpose** | **Gene** | **Forward Primer** | **Reverse Primer** |
| --- | --- | --- | --- |
| Transformation | *AvBADH* | gagaacacgggggactctagaATGTCGATCGCGATCCCGATTC | gcccttgctcaccatggatccTCAAAGCTTTGAAGGAGATTG |
| Detection | *35s* promoter | AAACCTCCTCGGATTCCATT | CGTGGTTGGAACGTCTTCTT |
| RT-qPCR | *AvBADH* | TATGGTCTAGGTGCTGCTGTGAT | GCTTCACGCTCAAGTAGTTCTCA |
